# Effect of Varying Levels of Glare on Contrast Sensitivity Measurements of Young Healthy Individuals Under Photopic and Mesopic Vision

**DOI:** 10.3389/fpsyg.2018.00899

**Published:** 2018-06-14

**Authors:** Marcello Maniglia, Steven M. Thurman, Aaron R. Seitz, Pinakin G. Davey

**Affiliations:** ^1^Department of Psychology, University of California, Riverside, Riverside, CA, United States; ^2^U.S. Army Research Laboratory, Human Research and Engineering Directorate, Aberdeen Proving Ground, Aberdeen, MD, United States; ^3^College of Optometry, Western University of Health Sciences, Pomona, CA, United States

**Keywords:** contrast sensitivity function, glare effect, mesopic vision, photopic vision, visual function measurement

## Abstract

Contrast sensitivity (CS), the ability to detect small spatial changes of luminance, is a fundamental aspect of vision. However, while visual acuity is commonly measured in eye clinics, CS is often not assessed. At issue is that tests of CS are not highly standardized in the field and that, in many cases, optotypes used are not sensitive enough to measure graduations of performance and visual abilities within the normal range. Here, in order to develop more sensitive measures of CS, we examined how CS is affected by different combinations of glare and ambient lighting in young healthy participants. We found that low levels of glare have a relatively small impact on vision under both photopic and mesopic conditions, while higher levels had significantly greater consequences on CS under mesopic conditions. Importantly, we found that the amount of glare induced by a standard built-in system (69 lux) was insufficient to induce CS reduction, but increasing to 125 lux with a custom system did cause a significant reduction and shift of CS in healthy individuals. This research provides important data that can help guide the use of CS measures that yield more sensitivity to characterize visual processing abilities in a variety of populations with ecological validity for non-ideal viewing conditions such as night time driving.

## Introduction

Vision represents our main modality of perception and interaction with the surrounding environment. While visual acuity (VA) is often considered the gold standard for vision assessment, contrast sensitivity (CS), defined as the ability to detect or discriminate low contrast gratings, may provide a more informative index of functional vision in both clinical and healthy populations (Owsley, 2003; Ng, 2012). For example, healthy individuals and patients can often reach the smallest font in a VA chart, but still report functional disabilities of vision that often coincide with a reduction of CS (Regan and Neima, 1984; Della Sala et al., 1985; Sokol et al., 1985). Thus a single clinical measure such as VA cannot adequately capture the full range of visual functional abilities, nor was it really designed to do so.

To obtain a broad-spectrum characterization of CS across multiple scales and frequencies, several clinical and laboratory tests have been developed to measure the CS function (CSF). While the CSF is generally measured in a low light environment, changing ambient lighting conditions can affect CS measurements in distinct and clinically relevant ways (Rabin, 1994; Pesudovs et al., 2004). Recently Safi et al. (2017) reported that the observed reduction of CS in diabetic patients depended on the illumination conditions of the testing stimulus; in particular, CS was impaired in response to spatial frequencies above 3 cycles per degree in photopic condition, while CS was reduced over the entire spatial frequency range in mesopic condition. Importantly, Safi et al. (2017) demonstrated better classification of diabetic patients from controls using a logistic model that incorporated measurements from two different light conditions compared to using a single CS measurement.

The CSF is impacted not just by changes in ambient lighting, but also by different sources of light that can interact with the optics of the eye to induce functional vision disability due to a phenomenon called glare. For example, upon leaving a dark room to go outdoors, one can experience a period of impaired vision due to the bright daylight, in what is called adaptation or discomfort glare. Another type of glare is the so-called disability glare, which is due to excessive exposure to light under dark conditions (i.e., night time driving) or to the scatter of light rays due to increasing ocular opacity (i.e., cataracts), which can impair functional vision and reduce sensitivity to contrast (Hrynchak, 1992), posing potentially serious hazard in daily life situations.

In this latter case, the scattered light reaches the retina in a sub-optimal way, creating a veil of straylight over the whole surface of the retina that adds to the image projection, reducing the retinal contrast and preventing the formation of a correct image (Vos, 1984). Retinal straylight emerges roughly 1° outside the point-spread function (Van Den Berg, 1995; Franssen et al., 2006) and its prominence increases with age (IJspeert et al., 1990) and with visual conditions such as retinitis pigmentosa (Alexander et al., 1996) or cataracts (De Waard et al., 1992). Glare is also a possible complication following cataract and refractive surgery: both photorefractive keratectomy (PRK) and laser-assisted *in situ* keratomileusis (LASIK) surgery (Seiler and Wollensak, 1991; Lohmann et al., 1993; Pérez-Santonja et al., 1998; Tahzib et al., 2005) have been linked with disability glare. Other causes of glare can include corneal diseases (Van Der Meulen et al., 2011) and excessive floaters in the vitreous humor (Mura et al., 2011).

Yet, studies in healthy individuals have shown that despite normal vision in daylight conditions, they may also exhibit serious impairment to vision under glare conditions (i.e., night driving, Anderson and Holliday, 1995), negatively affecting their driving performance (Ranney et al., 2000; Theeuwes et al., 2002). Indeed, glare produced by the headlights of an approaching car at night-time can reduce CS of a factor of 6 (Anderson and Holliday, 1995). Similarly, studies with cataract simulation in normal sighted individuals during everyday tasks such as reading speed and face recognition showed that CS was reduced with glare (Elliott et al., 1996).

In general, glare is a common visual complaint that impacts vision in both patients and normal sighted individuals, but standard acuity tests do not seem able to predict vision under non-ideal viewing conditions such as glare (Haegerstrom-Portnoy et al., 2000; Brabyn et al., 2001). Because of this, in order to produce a complete description of one’s visual functions, vision should be evaluated across different lighting and viewing conditions. Assessment under artificially induced glare in particular would provide a series of benefits: (i) it could be used as a tool to test vision in ecologically realistic scenarios (i.e., night-driving), (ii) it can provide a more sensitive assessment test for post-surgery visual evaluation, and (iii) it can expand the dynamic range of the CSF tests already employed in the clinic by reducing the baseline CS to avoid ceiling effects in young healthy individuals. Currently, no gold standard exists for assessing visual functions under glare conditions in healthy individuals.

A common CS measurement system in clinics, the CSV-1000E, allows for measuring CSF while simulating glare with the use of halogen lights producing an adjustable illumination intensity up to 69 lux (a standard measure of luminance, defined as the amount of light from a uniform source on a surface 1 m in radius). Using this device, Tuan and Liang (2006) reported a significant reduction of mesopic CSF under the glare-inducing condition for myopic astigmatism patients who underwent wavefront-guided LASIK procedures. In other studies with both cataract patients (Williamson et al., 1992) and normal sighted individuals from various age groups (Mahjoob et al., 2016), results showed a significant decrease in CSF under a lighting level of 4000 and 2000 lux, respectively, roughly 60–30 times higher than the intensity offered by the CSV-1000E. However, no study to date has documented both photopic and mesopic CSF measurements across a range of glare conditions in young healthy individuals, and it is unclear what exact level of light intensity would be necessary and sufficient to reliably induce glare in this population.

In the present paper, we first measured the photopic CSF of a group of young healthy individuals with and without glare for the standard luminance level provided by the CSV-1000E (Experiment 1). In Experiment 2, we measured the mesopic CSF of another group of young healthy individuals with the CSV-1000E with and without glare. The rationale behind Experiment 2 was that mesopic condition resembles a number of possible real-life situations such as night driving, thus representing an ecologically informative scenario. The results from these first two comparisons showed that the luminance level offered by the CSV-1000E is not sufficient to induce a statistically significant reduction of the CSF in photopic or in mesopic viewing condition in young healthy participants. Participants in Experiment 2 were also tested under mesopic light condition at a higher luminance level (225 lux), obtained with a custom-built glare-inducing system, and results revealed that the CSF was significantly reduced at this elevated luminance level.

Finally, as a follow up we tested a subgroup of participants from Experiment 2 on four additional intermediate luminance levels (125, 150, 175, 200 lux) and found that illumination levels of at least 125 lux were sufficient to induce significant glare and a reduction of mesopic CSF in healthy participants. Overall, these results suggest that under both photopic and mesopic conditions, the CSV-1000E cannot induce glare that significantly impairs CS in young healthy individuals. However, the use of higher luminance glare, obtained with our custom-built apparatus, was capable of significantly reducing CSF where 125 lux was found to be the sufficient level of intensity needed to reduce CSF in most individuals – a light intensity that is substantially higher than offered by current standard commercial tests.

## Materials and Methods

### Apparatus

**FIGURE 1:**
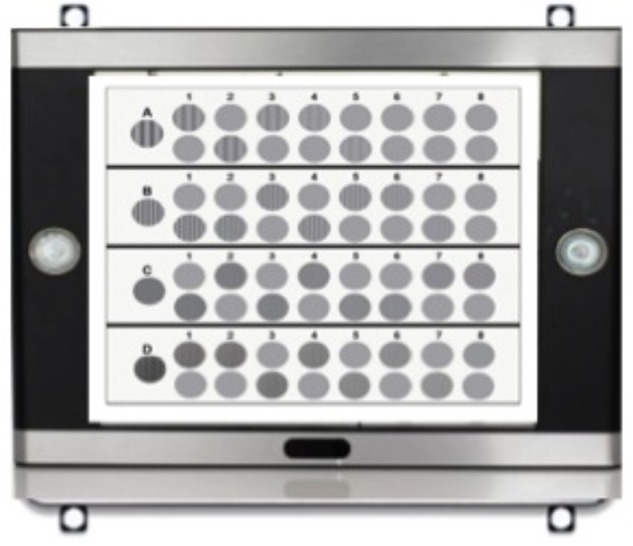
Custom modified CSV-1000E contrast sensitivity test with LED lights placed at each of the four corner of the table and oriented toward the observer.

A VectorVision CSV-1000E (Greenville, OH, United States) CS unit was used for the test. The CSV-1000E is a translucent retro-illuminated chart that consists of a series of circular achromatic sine-wave patches. The unit self-calibrates to produce a mean luminance of 85 cd/m^2^. CSF measurement was conducted under photopic condition (luminance level 10 to 10^8^cd/m^2^) for Experiment 1 and under mesopic (luminance level 0.001 to 3 cd/m^2^) in Experiment 2 and 3. Two glare conditions were tested in Experiment 1: no glare (standard CSF measurement) and glare (69 lux halogen light source). Experiment 2 utilized a custom built glare system that had four led light placed at the each corner of the device and angled to face to participant that was viewing the CS unit (see **Figure 1**). Three glare conditions were tested in Experiment 2: no glare, low glare (69 *lux* LED light source), and high glare (228 *lux* LED light source). In Experiment 3, a subgroup of participants of Experiment 2 were tested on four additional glare levels (125, 150, 175, and 200 *lux*), to better characterize the modulation of CSF induced by different levels of glare intensity. Mesopic vision was obtained with 1.5 neutral density filters.

### Participants

53 participants (19 males, mean age = 27.6 years) took part in Experiment 1, and 71 (21 males, mean age = 31.23 years) in Experiment 2. A subgroup of 10 participants from Experiment 2 took part in Experiment 3. All participants were recruited at Western University of Health Sciences, Pomona CA. The study was approved by the Institutional Review Board and all participants provided written informed consent to participate in the study. The study was conducted in accordance with the Declaration of Helsinki (1964). The data management was adhered with Health Insurance Portability and Accountability Act (HIPAA) regulations. All participants had normal or corrected-to-normal vision of 20/20 or better on ETDRS acuity chart. They took part in two testing session of about 30 min each.

### Procedure

Participants were tested at a distance of 2.5 m from the CSV-1000E. This distance allowed measuring spatial frequencies of 3, 6, 12, and 18 cycles per degree (cpd). The CSV-1000E is composed of a series of circular achromatic sine-wave patches 1.5′ in diameter. Across each row, there are vertical pairs of circles, one of which contains the sine-wave patch while the other is blank but has the same luminance as the test patch. There are four rows, each corresponding to one of four spatial frequencies. When selected, a given spatial frequency is rear-illuminated and a suprathreshold example of the test pattern is shown to the participant. Each spatial frequency is presented at eight different contrast levels that systematically decrease from 0.045 to 2.00, 0.7 to 2.20, 0.78 to 2.26, 0.6 to 2.08 and 0.3 to 1.81, respectively, in eight columns from left to right. The participant is asked to indicate whether the given test pattern is located in the top or bottom patch. The contrast threshold is defined as the contrast of the last column the subject could correctly identify the location of the sine-wave patch. The average inter-stimulus drop in contrast is 0.15 log units between steps 2 and 8; the contrast change between step 1 and step 2 is 0.3 log units.

## Results

**FIGURE 2:**
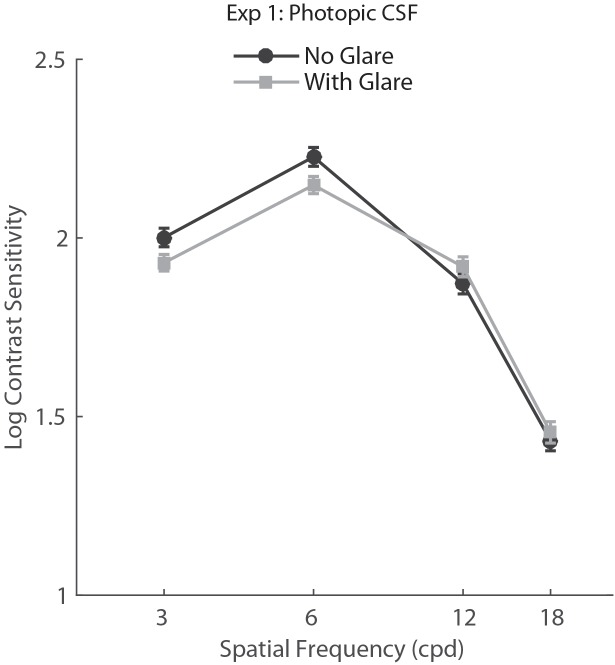
Contrast sensitivity function in photopic viewing for glare and no glare condition (69 lux) tested with CSV-1000E. Error bars represent standard error (SEM).

For Experiment 1, we conducted a two-way repeated measures ANOVA with factors Glare condition (Glare vs. no glare) and Spatial Frequency (3, 6, 12, 18 cpd). Results showed a main effect of Spatial Frequency (*F*_3,150_ = 427.16, *p*<0.001, η = 0.895) but no effect of glare condition on CS (*F*_1,50_ = 1.5, p = 0.227, η = 0.029), demonstrating there was no statistically significant difference between CS measurements obtained with and without glare under photopic conditions (**Figure 2**).

For Experiment 2, we conducted a two-way repeated measure ANOVA with factors Glare condition (no glare, low glare, high glare) and Spatial Frequency (3, 6, 12, 18 cpd). Results showed a main effect of Glare condition (*F*_2,148_ = 32.06, *p*<.001, η = 0.302), Spatial Frequency (*F*_3,222_ = 533.82, *p*<.001, η = 0.878) and a significant interaction Glare condition × Spatial Frequency (*F*_6,444_ = 3.125, *p*= 0.005, η = 0.041). *Post hoc* tests (Holm–Bonferroni-corrected *t*-tests) showed that the CSF in the high glare condition was significantly different (lower) from both no glare and low glare conditions (both *p*<0.001).

**FIGURE 3:**
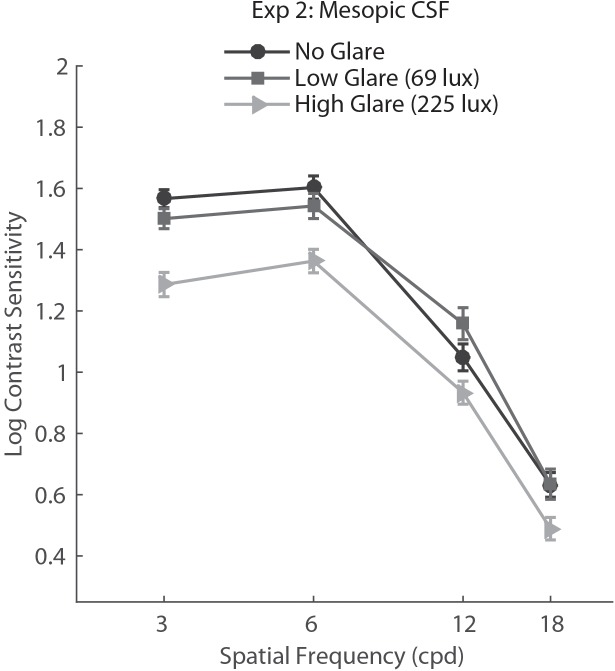
Contrast sensitivity function in mesopic light condition for no glare, low glare (69 lux), and high glare (228 lux) levels, tested with the custom-built setup. Error bars represent standard error.

*Post hoc* analysis on the Glare condition × Spatial Frequency interaction showed that the high glare group had significantly lower CS with respect to both low glare and no glare for all the SF tested (*p* values between 0.0001 and 0.024), while there was no difference between no glare and low glare condition for any of the SF (**Figure 3**).

In order to better understand the effect of glare on SF, we performed an additional repeated measure ANOVA on the difference between baseline and low glare (No glare score–Low glare score) and baseline and high glare (No glare score–High glare score). Alongside the main effect of Glare level (High vs. Low, F_1,74_ = 46.04, *p*<.0001, η = 0.384), the results showed a main effect of SF (*F*_3,222_ = 5.46, *p*= 0.001, η = 0.69), meaning that glare affected differently the various SFs tested.

**FIGURE 4:**
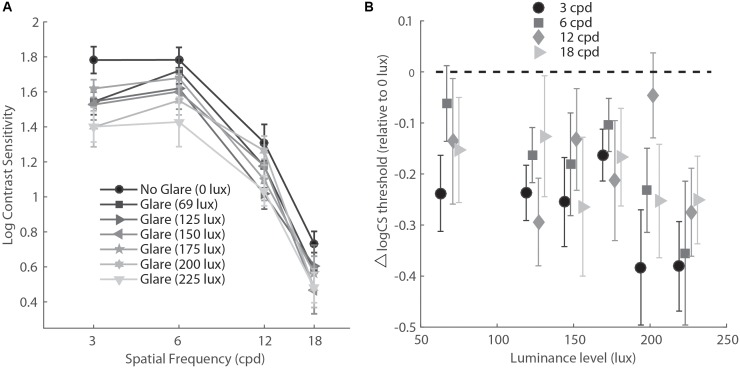
**(A)** Contrast sensitivity function in mesopic light condition for six levels of glare (variable lux) and no glare (0 lux). **(B)** Data re-plotted to show the difference between CS measurements at various levels of glare (abscissa) compared to no glare (glare – no glare condition), for each level of spatial frequency. cpd, cycles per degree.

To further explore the effect of glare on SF, we ran Holm–Bonferroni corrected multiple comparisons between the SF levels for the No glare-High glare differences. This test showed that the difference between high glare and baseline for the lowest SF (3 cpd) was significantly larger than for the higher spatial frequencies (*p* = 0.002 and *p* = 0.05 for 3 cpd vs. 12 cpd and 3 cpd vs. 16 cpd, respectively), meaning that glare reduced CS significantly more at lower spatial frequency than higher (**Figure 4**).

Finally, for Experiment 3, we conducted a two-way repeated measure ANOVA with glare condition (No glare, 69 lux, 125 lux, 150 lux, 175 lux, 200 lux, 225 lux) and Spatial Frequency (3, 6, 12, 18 cpd) as factors. Results showed a main effect of glare condition (*F*_6,54_ = 4.17, *p*= 0.002, η = 0.317) and Spatial Frequency (*F*_3,27_ = 146.91, *p*<.001, η = 0.942). *Post hoc* tests (Holm–Bonferroni-corrected *t*-tests) showed that except for the lowest level of glare (69 lux, corrected p = 0.067), all the other intensities induced a significant reduction of CS with respect to the no glare condition (corrected *p* = 0.03, 0.049, 0.049, 0.048, 0.014 for 69 lux, 125 lux, 150 lux, 175 lux, 200 lux, and 225 lux, respectively). While all light intensities greater than 69 lux induced a general reduction of CS measurements, the greatest reduction across all spatial frequencies was observed for the highest intensity level (225 lux).

## Discussion

In the present study, we measured CSFs in normal sighted participants under different light conditions and levels of glare, comparing a commercially available apparatus for CSF measurement (CSV-1000E) with a custom-built setup. In Experiment 1, we tested the CSV-1000E under glare condition, while in Experiment 2 and 3 we used the custom-built glare-inducing system that allowed for higher luminance levels compared to the CSV-1000E. Results showed that the level of glare induced by the latter (69 lux) was insufficient to affect the CSF under photopic (Experiment 1), and mesopic viewing conditions (Experiment 2) in young healthy participants. However, a higher level of luminance intensity (225 lux) under mesopic viewing condition reduced CS significantly at all the spatial frequencies tested (Experiment 2), and especially at lower spatial frequencies (3 cpd). A further experiment (Experiment 3), aimed at measuring a range of illumination intensities in mesopic light condition confirmed results obtained in Experiment 2 and showed that values above 125 lux did induce a significant decrease of the CSF. Taken together, the present results suggest that, while glare-induced reduction of the CSF can be achieved with currently available systems in clinical populations, i.e., astigmatism patients who underwent LASIK surgery (Safi et al., 2017), in order to successfully reduce the CSF in young healthy individuals it is necessary to use a luminance level that the currently available standard CS tests do not reach (125 lux).

The evidence that the relatively low intensity of luminance offered by the CSV-1000E induces glare in post-surgery patients (Tuan and Liang, 2006) but not in young healthy observers is certainly not surprising. Disability glare is known to affect patients and elderly individuals more than young, healthy ones. Indeed, our aim in the present study was to determine the luminance intensity that would allow to simulate the observed reduction of CS under glare in natural scenarios in a wider range of observers.

As stated in the introduction, reducing CS with glare presents a number of advantages: On one hand, it would allow to simulate a series of real life scenarios in which glare disrupts vision (i.e., night driving); on the other hand, it would provide a more sensitive assessment tool for the visual evaluation of patients recovering from eye surgery. Additionally, reducing the baseline CSF would increase the dynamic range of the test and reduce the influence of ceiling effects to measure subtle changes in groups with an already high-level of vision (e.g., athletes, young healthy individuals). Current market offers VA and CS tests that, while integrating artificially inducing glare equipment, do not seem to be effective in a healthy population.

Indeed, CS tests like the CSV-1000E offer a limited number of contrast levels per SF, therefore a young healthy observer or an athlete who wants to monitor his visual abilities might reach the lowest level of contrast provided by the test already during baseline measurements. Consistently, 3 out of 10 participants in Experiment 3 reached the lowest contrast value for 3 cpd already in the first measurement. A procedure that reduces the observer’s initial CSF would then provide a larger number of contrast values beyond his threshold, allowing to measure performance improvements beyond a level that without glare would be at ceiling.

Participants in Experiment 2 and 3 were tested under mesopic vision. The rationale was that in both normal sighted individuals and patients, it seems to be the light condition under which the CSF is mostly affected (Owsley, 1994), in particular patients who underwent corneal refractive surgery complain of night vision disturbances and glare despite normal VA (Brunette et al., 2000; McGhee et al., 2000). Indeed, under photopic condition the visual system relies mainly on cones and contrast thresholds are fairly constant across luminance levels (following Weber’s law, Walraven et al., 1990), while under mesopic vision light perception arises from a combination of cones and rods, resulting in luminance-dependent contrast thresholds, i.e., smaller changes in contrast at low luminance (Conifort et al., 1946).

This is due to the fact that under cone vision, gain control mechanisms maintain perceptual constancy for contrast over a wide range of illumination levels, avoiding response saturation (Shapley and Enroth-Cugell, 1984; Rutherford, 1987). On the other hand, rod vision favors sensitivity, therefore abandons gain control mechanisms and follows the deVries–Rose law: increment threshold is proportional to the square root of the background illumination (Rose, 1948). Mesopic vision is a combination of cone and rod vision, therefore it follows a pattern in between deVries–Rose and Weber behavior (Kang et al., 2009). Additionally, intraocular light scatter induces an increase in luminance in the regions of low lightness of the retinal projection, reducing proportionally as the image lightness approaches that of the scene (Stiehl et al., 1983); therefore straylight is likely to affect CS when it is above the mean luminance of the stimulus, as it is the case in mesopic vision. Taken together, these observations can explain the results reported here of CS reduction under glare in mesopic vision.

The practical applications of simulating glare under this lighting condition appear evident when one considers how both the rate (Williams, 2003; Bella et al., 2014) and the severity (Plainis and Murray, 2002) of fatal accidents is higher at night than in daylight and how the glare produced by the headlights of a car in this light condition can dramatically reduce CS (Anderson and Holliday, 1995). Indeed, simulating glare in a lab setting has the potential to increase individual awareness of the risks associated with distance and speed perception disruption induced by glare. Nonetheless, future studies should also assess whether the photopic CSF can be similarly affected by extremely high levels of glare in young healthy individuals. This would provide an additional valuable piece of information since Safi et al. (2017) showed that comparing CS measured in different light conditions produces a more accurate classification of diabetic patients with no retinopathy from normal sighted individuals respect to the CS measured under a single light condition; similarly, comparing mesopic and photopic CS with glare might reveal subtle differences that might be lost in a single light condition test.

## Conclusion

In conclusion, it is possible to significantly reduce the CSF of young healthy individuals using artificially induced glare; however, this requires a level of luminance that is about 2–3 times higher than what CS tests currently offer on the market today. Of note, we nonetheless showed that it is possible to induce a CSF reduction with an illumination intensity 10 times lower than what has been proven to be effective in a previous studies (125 lux vs. 2000 lux and 4000 lux; Williamson et al., 1992; Mahjoob et al., 2016). Glare is an ecologically valid model, in that it pertains to a number of real life lighting conditions for both normal and clinical populations, often overlooked in standard CS tests (for example glare is a parameter for visual function that is not evaluated prior to obtaining a driver’s license), and it also represents a potential improvement over classic CSF measurements, in that it can expand the dynamic range of the standard tests and avoid the common issue of ceiling effects in clinical CS tests (Koefoed et al., 2015; Wang et al., 2015). Further research needs to examine the diagnostic value of these measures and their implementation in standard visual assessments in clinical practice for both patients and individuals with normal or above average visual abilities.

## Author Contributions

PGD designed and implemented the experiments, and data was collected by the technicians in his lab. MM analyzed the data. All authors interpreted the results, and wrote and reviewed the main manuscript.

## Conflict of Interest Statement

PGD has received funding support and is a consultant to VectorVision not related to present study. The remaining authors declare that the research was conducted in the absence of any commercial or financial relationships that could be construed as a potential conflict of interest.
